# Ten-year trends in antimicrobial and analgesic prescribing by NHS dentists in England: a retrospective analysis study

**DOI:** 10.1038/s41415-026-9787-3

**Published:** 2026-07-10

**Authors:** Nikul Patel, Noha Seoudi

**Affiliations:** 418992895561372969034 Department of Oral Microbiology, University Dental Hospital, Cardiff, United Kingdom; 373374846641003471752 Institute of Dentistry, Queen Mary University of London, London, UK; Barts Health Dental Hospital, Barts Health NHS Trust, London, UK; College of Medicine and Dentistry Outreach Centre of Ulster University, Birmingham, United Kingdom

## Abstract

**Aims** Evidence on long-term National Health Service (NHS) dental prescribing is limited. This study aimed to identify whether utilisation of analgesics and antimicrobials returned to pre-Coronavirus Disease 2019 (COVID-19) levels in primary dental care, highlighting the national efforts needed to ensure patient safety when prescribing in dentistry.

**Methods** Ten years of data from the NHS Business Services Authority on the utilisation of antimicrobials and analgesics by primary care dentists between January 2014 and December 2023 were obtained. Significant distributional changes were analysed using chi-square test, focusing on the impact of COVID-19 restrictions on prescribing.

**Results** Total antimicrobial and analgesic prescribing returned to near pre-pandemic levels by December 2023 (antimicrobials – February 2020: 373.8, December 2023: 358 items/100,000; analgesics – February 2020: 8.78, December 2023: 8.35 items/100,000). Amoxicillin, metronidazole, erythromycin, paracetamol and diclofenac fell significantly versus peak (*p* <0.05). Clindamycin declined post-pandemic but remained above pre-pandemic levels (February 2020: 1.83, December 2023: 2.00 items/100,000). Dihydrocodeine comprised 42% of analgesics in December 2023 and exceeded pre-pandemic levels (February 2020: 3.22, December 2023: 3.51 items/100,000).

**Conclusions** Prescribing guidelines and stewardship programs contributed to reduced antimicrobial use, though pandemic disruptions reversed progress. Relatively higher clindamycin prescribing raises concerns about *Clostridioides difficile* risk. Continued reliance on dihydrocodeine despite its side-effect profile highlights the importance of a national plan to ensure regular training and self-audit.

**Supplementary Information:**

Zusatzmaterial online: Zu diesem Beitrag sind unter 10.1038/s41415-026-9787-3 für autorisierte Leser zusätzliche Dateien abrufbar.

## Key points

- Evidence on long-term NHS dental prescribing is limited; this ten-year study evaluates trends, stewardship impact and whether prescribing returned to pre-pandemic levels following COVID-19 dental service disruptions.
- Prescribing declined over ten years but increased during COVID-19 restrictions; clindamycin and dihydrocodeine remained above pre-pandemic levels despite limited clinical justification and safety concerns.
- Targeted stewardship, training and self-audit are needed to improve prescribing safety, reduce inappropriate antimicrobial and opioid use, and support national efforts to combat antimicrobial resistance in dentistry.

**Supplementary Information:**

Zusatzmaterial online: Zu diesem Beitrag sind unter 10.1038/s41415-026-9787-3 für autorisierte Leser zusätzliche Dateien abrufbar.

## Introduction

Antimicrobial resistance (AMR) is a global health issue, often described as a silent pandemic.^1^ The World Health Organization (WHO) has declared AMR a global emergency, and ranked AMR among the top ten global public health threats.^2^^,^^3^ By 2050, AMR is projected to cause ten million deaths annually and 28.3 million people could be forced into extreme poverty with an estimated economic burden of $100 trillion.^4^^,^^5^^,^^6^ Alarmingly, global antibiotic consumption is expected to triple by 2030.^6^

In the UK, primary care accounts for 80% of all National Health Service (NHS) antibiotic prescriptions,^7^ with primary care dental services contributing approximately 10%.^8^ Dental services do not have access to clinical oral microbiology support, resulting in limited susceptibility data and poor surveillance for resistance in dental infections. Hospitals by contrast use broad spectrum agents for complex cases but benefit from clinical microbiology services and multidisciplinary antimicrobial stewardship (AMS) programmes. Antimicrobials in dentistry should be reserved for dentoalveolar infections with signs of spread of infection, with surgical intervention as the principal management.^9^ The use of antimicrobials carries risks of adverse reactions and accelerates AMR. Evidence suggests that dentists may prescribe antimicrobial agents inappropriately for dental pain, when surgical means are unavailable or when under patient pressure.^10^

The FDI World Dental Federation has actively raised awareness about AMS in dentistry.^11^ The FDI encourages dentists to align their prescribing practices with best practices and promotes educational programmes on AMR and AMS throughout dental professionals' careers.^12^ Additionally, it supports national dental associations in implementing AMS programmes within national action plans to combat AMR.^11^

Since 2015, various working groups, such as English Surveillance Programme for Antimicrobial Utilisation and Resistance (ESPAUR) dental subgroup, have been set up to address antimicrobial usage in dentistry by developing toolkits specific for AMS in dentistry.^13^ Furthermore, multiple UK national guidelines were developed, such as the *Antimicrobial Prescribing in Dentistry: Good Practice Guidelines*, to recommend management of dental infections and appropriate use of antimicrobial agents. This guideline is the outcome of collaboration between the Faculty of General Dental Practitioners (FGDP, currently College of General Dentistry) and Faculty of Dental Surgery, Royal College of Surgeons of England. In Scotland, the Scottish Dental Clinical Effectiveness Programme (SDCEP) has also developed a guideline for drug prescribing.^9^^,^^14^

Other countries have also made significant strides in AMS in dentistry. For instance, governmental strategies in Sweden to reduce antimicrobial usage in dentistry, starting in 2012 and 2014, led to a marked reduction in antimicrobial prescriptions from dentists.^15^ Sweden's approach is part of a broader national strategy that emphasises a ‘one health' perspective, which recognises the interconnectedness of human, animal, and environmental health.^16^ This holistic approach has led to continued success in promoting rational antimicrobial use in dentistry. The Swedish Strategic Programme for Rational Use of Antimicrobial Agents has been a key driver in these efforts since 1995, serving as a potential model for other countries in implementing effective AMS in dental practice.^15^

Between 2010 and 2017, UK antibiotic prescribing declined significantly, with overall primary care prescribing falling by 14.8% and dental antibiotic prescribing decreasing by 24.4%.^17^ Dental prescribing accounted for 10.8% of all NHS oral antimicrobial prescribing in England, which was higher than Australia's 3% but lower than Canada's 11.8% and the USA's 13.2%.^17^ In England and Scotland, amoxicillin and metronidazole comprised 64.8% and 28.0% of dental prescriptions, while in Sweden and Norway, Phenoxymethylpenicillin dominated at 80.37% and 68.49%, respectively, in 2010.^18^

The Coronavirus Disease 2019 (COVID-19) restrictions disrupted this downward trend driving an increase in remote prescribing.^19^^,^^20^ Guidelines instructed triage through telephone or virtual consultations and to provide advice, analgesia and antimicrobials when appropriate. International impacts varied. France saw an 18.2% reduction in 2020 and in Australia, the total dental antibiotic prescriptions decreased, but antibiotics accounted for a higher proportion of dental prescriptions.^21^^,^^22^ In Alberta, Canada, prescribing surged by 76%, mirroring rises in England and Scotland.^23^ Scotland recorded a 49% increase between June 2019 and June 2020, and England, a 25% rise from April to July 2020 versus 2019 (although declined after these peaks, they remained above pre-pandemic levels). Scotland averaged 17.3 antibiotic items per 100 claims between December 2020 and May 2021, compared to 5.4 pre-pandemic, and England recorded 5.3 prescriptions per 1,000 population in July 2020, up from 3.9 in 2019.^20^^,^^24^ Recent data confirm persistent elevation across all UK nations, with cumulative dispensing from March 2020 to August 2023 at 175.6 in England, 227.2 in Scotland, 195.0 in Wales, and 321.8 in Northern Ireland per 1,000 population – excess of 27.7%, 43.3%, 33.2%, and 42.9%, respectively, compared to predicted trends.^25^

During the COVID-19 restrictions on dental care in England, analgesic prescriptions increased, with the total increasing from 15,507 prescriptions between April and June 2019 to 28,563 prescriptions in the same months of 2020.^19^ Dihydrocodeine prescribing saw a significant rise, accounting for 40.91% of total prescriptions in 2020, compared to 32.91% in 2019.^19^ Similarly, Diclofenac prescribing increased to 24.63% of total prescriptions, up from 12.77%.^19^ In contrast, Australia recorded an 18% drop in opioid prescriptions in April 2020 compared to April 2019.^22^ However, there was an increase in certain opioids later during the pandemic, with Tramadol prescriptions rising by 11% in May and 46% in June 2020 compared to the same months in 2019. Oxycodone prescriptions also saw a consistent increase across all months of 2020, except for April, with an average rise of 22% compared to 2019. Diclofenac prescriptions in Australia decreased by 42% in April and 29% in May 2020 compared to the same months in 2019.^22^

Previous UK studies have examined short-term antibiotic trends or COVID-19 surges, but none have jointly analysed a decade of antimicrobial and analgesic prescribing, tested post-pandemic reversion, or focused on safety-critical classes. This study addresses these gaps by analysing NHS dental prescribing data from 2014–2023 to evaluate long-term trends, the impact of COVID-19 restrictions, and the persistence of relatively high prescribing rates of high-risk drug classes.

## Materials and methods

A Freedom of Information request (FOI-01901 and FOI-01126) for prescribing data from the NHS Business Services Authority was made for the period between January 2014 to December 2023 (ten years). Prescribing data requested for NHS primary care dental services (general dental practices and primary care specialist services) on antimicrobial and analgesic items prescribed specifically:

- The monthly number of items of antibacterial agents in the British National Formulary (BNF) section 5.1 prescribed and dispensed in England by NHS general dental practitioners (GDPs) for the periods 1 January 2014 to 31 December 2023 expressed as BNF substance code, chemical names, and number of items
- The monthly number of items of analgesics (paracetamol, aspirin, dihydrocodeine, diclofenac sodium and ibuprofen) prescribed by NHS GDPs for the periods 1 January 2014 to 31 December 2023 expressed as BNF substance code, chemical names, and number of items
- Drug items were combined based on their primary drug name (e.g., doxycycline hyclate and doxycycline monohydrate were combined to doxycycline)
- To aid the analysis of long-term trends in prescribing, comparison across different time periods and identify changes in prescribing behaviour items were calculated per 100,000 population to help mitigate the effect of population change.

All data received were analysed using Microsoft Excel and Data Analysis tool (Microsoft, USA). To identify whether prescribing during the peak period differed significantly from the overall post-restriction pattern, chi-square test of goodness-of-fit tests were applied to compare prescribing rates (items per 100,000 population) during the peak month of COVID-19 restrictions with rates from all other months between February 2020 and December 2023.

As this study involved the analysis of anonymised data obtained from the NHS Business Services Authority, and no patient-identifiable information was accessed and no direct contact with human participants occurred, obtaining ethical approval and consent was not required in accordance with institutional and national guidelines for research involving routinely collected administrative data.

## Results

Between 2014 and 2023, there was slight increase in England's population (54,370,300 to 57,690,300) and slight increase in the number of NHS dentists (23,947 to 24,193); however, the number of GDPs was highest in 2019 (24,684), and levels of NHS dentists have not returned to this level after COVID-19. Furthermore, the number of NHS dentists to 100,000 population has also decreased between 2014 and 2023 (44.04 dentists/100000 population to 41.93 dentists per 100,000 population, respectively).

Between January 2014 and December 2023 prescribing was 53,654.05 antimicrobial items per 100,000 population totalling 29,798896 items (online Supplementary Table 1). Penicillin-class antimicrobials were the most prescribed, accounting for 67.04% of all prescribed items (35,971.35 items per 100,000 population), with amoxicillin being the most prescribed antimicrobial at 65.9% of total antimicrobial items (35,357.25 items per 100,000). Amoxicillin accounted for 98.3% of total penicillin-class antimicrobials, followed by phenoxymethylpenicillin at 1% (352.50 items per 100,000 population) and co-amoxiclav at 0.3% (261.61 items per 100,000 population). The second most prescribed antimicrobial is metronidazole, the only nitroimidazole class with 28.44% of total antimicrobials prescribed (15,258.15 items per 100,000 population). The third most common class of antimicrobial prescribed was macrolides at 3.59% of total antimicrobials (1,926.28 items per 100,000 population). Erythromycin contributed to 89.5% to total macrolides prescribed (1,725.63 items per 100,000 population). Clindamycin contributed to 0.51% of total prescriptions over the ten-year period (274.97 items per 100,000 population). The total amount of antimicrobial items decreased from 2014 to 2023 (6,797.72 items per 100,000 to 53,654.05 items per 100,000, respectively). The total amount of antimicrobial items decreased from 2014 to 2023 (6,797.72 items per 100,000 to 4,346.48 items per 100,000). Across most individual antimicrobials, there was a decrease in items prescribed when comparing the years of 2014 and 2023 except for azithromycin (2014: 3.94; 2023: 5.17) clarithromycin (2014: 8.79; 2023: 18.89) and phenoxymethylpenicillin (2014: 50.40; 2023: 60.15).

**Fig. 1:**
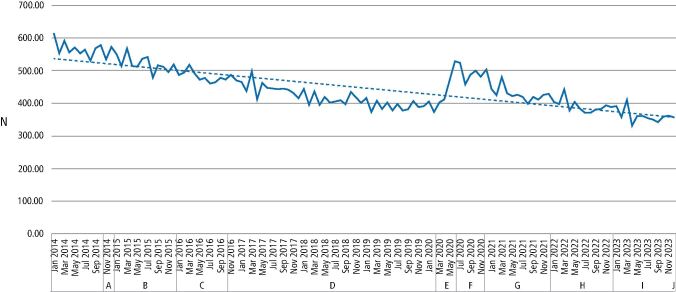
Monthly prescribing rates (items per 100,000 population) of combined antibiotics from January 2014 to December 2023 (Key: A = BDA Antimicrobial Summit in Dentistry; B = formation of ESPAUR dental subgroup; C = SDCEP Drug Prescribing Guidelines 3^rd^ Edition; D = Dental AMS Toolkit UK HSA; E = COVID-19 restrictions on dental practices; F = post-COVID-19 restrictions to dental care; G = FGDP Antimicrobial Prescribing Guidance 3^rd^ Edition; H = KAW and BSAC Dental Core Group formation; I = SDCEP drug prescribing website launched; J = update of Dental AMS Toolkit UK HSA)

**Fig. 2:**
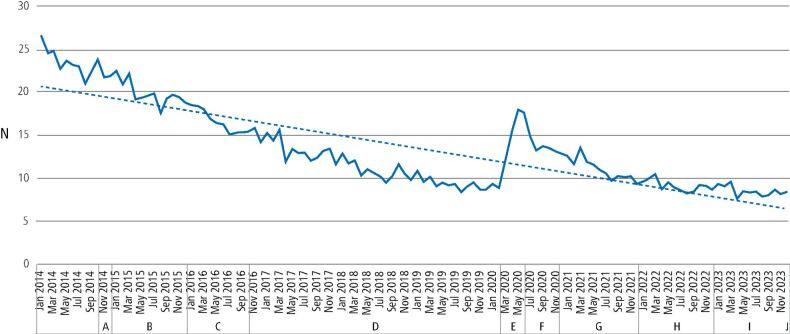
Monthly prescribing rates (items per 100,000 population) of combined analgesics from January 2014 to December 2023 (Key: A = BDA Antimicrobial Summit in Dentistry; B = formation of ESPAUR dental subgroup; C = SDCEP Drug Prescribing Guidelines 3^rd^ Edition; D = Dental AMS Toolkit UK HSA; E = COVID-19 restrictions on dental practices; F = post-COVID-19 restrictions to dental care; G = FGDP Antimicrobial Prescribing Guidance 3^rd^ Edition; H = KAW and BSAC Dental Core Group formation; I = SDCEP drug prescribing website launched; J = update of Dental AMS Toolkit UK HSA)

There was a general downward trend until 2020 (COVID-19 pandemic) when the antimicrobials prescribing increased sharply. The prescribing level then started to reduce again, reaching a level slightly below the pre-pandemic level (Fig. 1). The timeline trend in relation to the different contributing factors (AMS programme and COVID-19 pandemic dental service restriction), which might have influenced the antimicrobial and analgesics prescribing patterns positively or negatively, is illustrated in Figure 1 and Figure 2. Individually, seven antimicrobials had a peak during the restriction period of dental services during COVID-19 (March 2020 – June 2020). Phenoxymethylpenicillin utilisation had an increase in November 2020 and had continued to rise, with its highest prescribing rate in December 2023 (5.60 items per 100,000). Nine antimicrobials prescribing rates have reduced to below pandemic levels but azithromycin, clarithromycin, clindamycin, and phenoxymethylpenicillin prescribing rates have increased. Specifically, the prescribing rate of clindamycin in February 2020, the month before COVID-19 restrictions to dental services, was 1.83 items per 100,000 population. At the peak of the pandemic, prescribing of clindamycin reached 3.09 items per 100,000 units and in December 2023, the items prescribed of clindamycin is 2.00 items per 100,000 population, remaining higher than the month pre-pandemic restrictions.

In the same period, analgesic prescribing was 1,615.45 items per 100,000 population, totalling 899,671 items (see online Supplementary Table 1). Non-steroidal anti-inflammatory drugs (NSAIDs) were the most prescribed type of analgesic (862.78 items per 100,000; 53.41%). Ibuprofen was the most prescribed individual analgesic at 661.68 items per 100,000 population (40.96%), followed by dihydrocodeine (494.46 items/100,000 population; 30.61%) and paracetamol (258 items/100,000 population; 15.98%). Like the trends observed in antimicrobial prescribing, there has been a notable decline in analgesic prescribing from 2014 to 2020, preceding the onset of the COVID-19 pandemic. However, a significant increase is evident during May 2020 (17.9 items/100,000 population), corresponding with the imposition of restrictions on dental care. When comparing prescribing patterns from the month of February 2020, before the pandemic restrictions, to December 2023 (February 2020: 8.8 items/100,000 population; December 2023: 8.4 items per 100,000 population) the total prescribing rate has returned to levels comparable to those seen before the pandemic (Fig. 2).

In the month before the COVID-19 restrictions (February 2020), NSAIDs accounted for 49.6% of all analgesics prescribed by dentists (total: l4.26 items per 100,000 population). Specifically, ibuprofen was prescribed at a rate of 3.4 items per 100,000, while diclofenac was prescribed at a rate of 0.96 items per 100,000. In comparison to January 2014, this represents a decrease in the proportion of NSAIDs within the total analgesic prescriptions, where they contributed 60.6% (a total of 16.0 items per 100,000 population). When examining dihydrocodeine prescribing, there was a reduction in the total number of items prescribed between January 2014 and February 2020, from 5.10 to 3.22 items per 100,000 population. However, its percentage contribution to overall analgesic prescribing increased from 19.2% in January 2014 to 36.7% in February 2020.

During the COVID-19 restrictions on dental care, prescribing rates for paracetamol, ibuprofen, dihydrocodeine, and diclofenac all increased, with monthly prescribing rates reaching 3.71, 3.65, 7.32, and 4.86 items per 100,000, respectively. By December 2023, the monthly prescribing rates for ibuprofen and paracetamol had returned to levels lower than those observed in February 2020 (paracetamol – February 2020: 1.20; December 2023: 0.89. ibuprofen – February 2020: 3.40; December 2023: 2.24). Prescribing of dihydrocodeine and diclofenac had decreased from their peak levels during the COVID-19 restrictions; although, both remained above the February 2020 prescribing rates. In December 2023, the dihydrocodeine prescribing rate was 3.81 items per 100,000, compared to 3.22 items per 100,000 in February 2020. Similarly, diclofenac prescribing in December 2023 was 1.71 items per 100,000, compared to 0.96 items per 100,000 in February 2020. In terms of the percentage contribution to total analgesics prescribed, dihydrocodeine accounted for 42.1% of all analgesics prescribed in December 2023, compared to 36.7% in February 2020, while NSAIDs accounted for 47.4% in December 2023, a decrease from 49.6% in February 2020.

In assessment of the peak prescribing month following the pandemic against the months between February 2020 and December 2023, amoxicillin, metronidazole, erythromycin, paracetamol and diclofenac showed significant differences (*p* <0.05, chi-squared), with reductions from peak values (online Supplementary Table 2).

## Discussion

This study complements and extends the knowledge gained from a recently published national analyses of dental prescribing during the COVID-19 pandemic conducted by Bowman-Newmark *et al*.^25^ The study used predictive modelling to estimate the excess antibiotic prescribing across the UK. The findings demonstrated that, as of August 2023, antibiotic prescribing rates in all four UK nations remained significantly above levels predicted from pre-pandemic trends, indicating that prescribing behaviours had not yet returned to expected baselines. This study similarly observed a disruption to the downward trajectory of antimicrobial prescribing in England before the pandemic dental restrictions. These findings are consistent with an earlier study, which reported increased antibiotic use during periods of restricted access to face-to-face dental care and reliance on remote triage protocols.^26^ Using February 2020 as a pre-restriction reference point, we found rates of antimicrobial prescribing in December 2023 was 358.36 items/100,000 and less than February 2020, which had a prescribing rate of 373.84 items/100,000, which indicates a return to pre-COVID-19 antimicrobial utilisation rate by the end of 2023 in England. However, deeper analysis of the data highlighted a concern with clindamycin utilisation which remained higher than the pre-COVID-19 utilisation rate. In addition to antimicrobial trends, our study uniquely explores analgesic prescribing. Similarly to antimicrobials, the total number of analgesics prescribed in December 2023 is 8.35 items/100,000, which is below February 2020 prescribing rate of 8.78 items/100,000. However, this study identified a sustained increase in dihydrocodeine use post-pandemic.

### Overall antimicrobial prescribing 2014–2023

Interventions have been implemented over the last ten years to raise awareness of AMR and to help reduce inappropriate antimicrobial usage. It is reported that 80% of antimicrobials prescribed by primary dental services to be inappropriate.^10^ There has been a 36.06% reduction in total antimicrobials prescribed in 2023 when compared to 2014. Within this period, the pandemic and restrictions to dental services saw an increase in antimicrobial prescriptions. Comparing the pre-pandemic year of 2019 to 2014, there was a 30.6% decrease in the yearly volume of items prescribed, suggesting the effects of the pandemic impacted the decreasing trend. During the COVID-19 restrictions between March 2020 and July 2020, dental practices were limited to urgent dental care only and were to perform a remote risk assessment and triage at the first stage. At this stage patients could be placed on the ‘Advice, Analgesia and Antimicrobial where Appropriate' pathway to be managed remotely in the first instance.^27^ The guidance acknowledged that efforts had been made to reduce inappropriate prescribing in dental practices but reasoned that surgical therapy may not be available, thus antimicrobials could be considered in cases of spreading infection, systemic involvement, or where immediate drainage was not possible.^28^

A previous study observed an 18.5% reduction in antimicrobials prescribed between 2012 and 2016.^8^ The British Dental Association led the Keep Antimicrobials Working Summit to establish an AMS programme in dentistry in November 2014. This was followed by establishing the ESPAUR dental subgroup to create and publish the first edition of the Dental Antimicrobial Stewardship Toolkit in November 2016 on the UK Health Security Agency website. Based on the prescribing rates alone, it is difficult to establish a direct causation and impact of the AMS programmes; however, before the pandemic, there was a steady decrease in primary care prescriptions of antimicrobials.^17^

Multiple publications highlighted the upward trend of antimicrobials and analgesics prescribing during COVID-19 dental care restriction.^19^^,^^20^^,^^24^ This led to the development of the dental core group of Keep Antibiotics Working to publish educational resources on the British Society of Antimicrobial Chemotherapy (BSAC) website in 2022. Furthermore, the Dental Antimicrobial Stewardship Toolkit was updated in December 2023 by the reassembled ESPAUR dental subgroup. Antimicrobial usage in dentistry should be seen as an adjunct to surgical intervention when justified if there are signs of spreading infection, sepsis, facial swelling or lymphadenopathy.^9^ These continuous national endeavours might have contributed to the reported reduction in the antimicrobials prescribing to the pre-COVID 19 prescribing in England.

### Individual antimicrobial prescribing trends

Amoxicillin is the most prescribed antimicrobial followed by metronidazole, erythromycin phenoxymethylpenicillin and clindamycin. Amoxicillin has traditionally been the antimicrobial of choice for acute dental infections when indicated and erythromycin and metronidazole as second-line alternatives, especially in view of penicillin allergy. Phenoxymethylpenicillin is recommended over amoxicillin because of its narrow spectrum of activity with less predicted dysbiosis in the microflora and the related complications.^9^^,^^14^ The Scottish Antimicrobial Prescribing Group released a statement in October 2020 recommending phenoxymethylpenicillin as the first-line antimicrobial for dental infections, which might have led to the observed increase in its prescribing.^29^ Macrolides including erythromycin, clarithromycin and azithromycin should only be used when justified, in cases with reported true penicillin allergy. Erythromycin is less preferred to clarithromycin in both FGDP and SDCEP guidelines, primarily due to its higher gastrointestinal side effects and compromised patient tolerance, both of which can negatively affect compliance. Having said this, clarithromycin has a broader spectrum of activity than erythromycin, including improved efficacy against gram-positive and certain gram-negative bacteria. This broader activity is not necessarily advantageous, as it may contribute to the disruption of the normal microflora. Resistance rate to the macrolide group is reported to be increasing; therefore, these are categorised in the ‘watch group' in the UK's antibiotic classification tool which is based on the WHO's Access, Watch or Reserve classification system.^30^ In England, clarithromycin prescribing has declined but remains above pre-pandemic levels. While specific data are lacking, this may be partly due to its continued use in patients with a reported penicillin allergy, for whom it is often prescribed as an alternative.^31^ However, there are no data to confirm increasing rates of true penicillin allergy. Therefore, this increase in rates of utilisation of macrolides is not justified and need to be addressed nationally.

The prescribing rate of clindamycin had decreased from January 2014 to February 2020, then a significant increase during COVID-19 was observed. The clindamycin prescribing rate after the pandemic restrictions remained higher than pre-pandemic levels. Though the trend is not statistically significant, the rate of clindamycin prescribing remains a substantial concern as safer alternative antimicrobials are available for patients with reported penicillin allergy. FGDP guidelines do not recommend the use of clindamycin and suggest it should be led by specialist in oral/medical microbiology.^9^ Furthermore, clindamycin is in the ‘reserve list', and their use should be limited to the cases when there is no effective alternative guided by specialists. Clindamycin has a high risk of significant morbidity and mortality due to the high rate of related *Clostridioides difficile* superinfection causing life-threatening pseudomembranous colitis; thus, patients should be made aware of the risk when given prescriptions.^32^ Although absolute UK prescribing volumes are low compared to countries where clindamycin use is linked to community *C. difficile*, its persistent elevation post-pandemic remains clinically relevant.^17^^,^^18^^,^^33^

### Overall analgesic prescribing 2014–2023

The trend of prescribed analgesic items reduced over the years before COVID-19, probably due to the availability of NSAIDs over the counter, but when restrictions were brought in for routine dental care, dentists were required to manage dental pain with prescribing or advising analgesia, with limited guidance.^19^ This study only includes data from NHS primary care dental prescriptions in England and does not include over-the-counter purchases of pain relief which often is cheaper to the patient and the NHS.

### Individual analgesic prescribing trends

Ibuprofen in 2014 was prescribed almost three times more that of paracetamol; however, over the ten years, the number of items of ibuprofen prescribed has greatly reduced, with little increase in the prescribing rate of ibuprofen during the pandemic. At the time, there was anecdotal evidence suggesting the use of NSAIDs, especially ibuprofen, could worsen the symptoms of COVID-19, but after thorough review, there was no association that ibuprofen led to increased mortality or need for increased respiratory support.^34^ Thus, the regime for analgesia was of paracetamol and/or ibuprofen in the first instance or replacing ibuprofen with diclofenac all with in recommended daily dosages.^28^

During the pandemic restrictions, diclofenac had a five-fold increase in prescribing between the month of February 2020 and its peak prescribing rate in June 2020, and remained higher during the post-pandemic period when compared to the month before the pandemic. Both diclofenac and ibuprofen work by inhibiting cyclooxygenase enzymes, reducing prostaglandin synthesis and inflammation which is effective in dental pain.^35^^,^^36^ A meta-analysis concluded there is no significant difference between ibuprofen and diclofenac in the treatment of dental pain; although, single studies highlighted a faster onset of action for severe pain.^37^^,^^38^ Ibuprofen remains the first-line NSAID due to a preferred safety profile and lower risk of side effects and cost.^34^

Dihydrocodeine is a synthetic opioid analgesic acting on opioid receptors in the central nervous system. The pandemic period observed a peak in dihydrocodeine prescribing and although decreasing has remained above pre-pandemic level, dihydrocodeine has increased its percentage contribution to 42.0% of analgesia prescribed in December 2023 from 36.7% in February 2020. There was no explicit mention of dihydrocodeine in management of dental pain and the BNF is clear in advising against the use for dental pain due to risks of addiction, constipation and nausea.^14^ Dihydrocodeine is in the dental formulary, but it is ineffective with dental pain, especially in comparison to ibuprofen, due to the lack of action to reduce inflammation.^34^^,^^39^ The increase in its percentage contribution and prescribing rate remaining high after the pandemic is a cause of concern to warrant further monitoring of opioid prescribing.

### Limitations

This study provides valuable insights into NHS dental prescribing patterns, with opportunities for further investigation. While it focuses on NHS prescribing data in England, data from private dental practices were not available and therefore not included, which may limit generalisability of the findings. Additionally, although the study captures prescribed analgesics data on broader pain management strategies, the use of over-the-counter analgesics within dentistry was not collected, and further research would be needed to explore this area comprehensively. Lastly, in alignment with international consensus on AMS outcomes, the study focuses on antimicrobial use, providing a foundation for future research into critical antibiotic use outcomes, patient-reported outcomes, and the impact of adverse events in dental care.^40^

## Conclusion

This study highlights key trends and areas of concern in prescribing practices for both analgesics and antimicrobials from 2014 to 2023 in England:

- Return to pre-pandemic utilisation levels – prescribing rates for both analgesics and antimicrobials have largely reverted to pre-pandemic levels following the COVID-19 restrictions in England NHS primary dental care
- Clindamycin use – clindamycin prescribing has not decreased to pre-pandemic levels, posing concerns due to its association with *C. difficile*-related colitis
- Opioid use – the continued use of dihydrocodeine and other opioids should be minimised given their side-effect profile and limited efficacy for dental pain
- Training and self-assessment – to support safe, sustainable and effective prescribing, there is a need for ongoing professional development through targeted training, education, and regular self-administered audits or peer review.

## Supplementary Information

Supplementary Tables 1-2 (PDF 224KB)

## Data Availability

The data used in this study were obtained from the NHS Business Services Authority through a formal data access request. These data are not publicly available but may be accessible to other researchers upon reasonable request and subject to approval by the NHS Business Services Authority.
