# Supplementary material for: Ten-year trends in antimicrobial and analgesic prescribing by NHS dentists in England: a retrospective analysis study
Source: Br Dent J. 2026 Jul 10;241(1):60–6. doi: 10.1038/s41415-026-9787-3 (PMC13354529; doi:10.1038/s41415-026-9787-3)
Supplement: Supplementary file 1 — Supplementary Tables 1-2 (PDF 224KB) [file 41415_2026_9787_MOESM1_ESM.pdf]

Drug	Year (Items/100000 population)											
**Antibiotic**	**2014**	**2015**	**2016**	**2017**	**2018**	**2019**	**2020**	**2021**	**2022**	**2023**	**Grand Total**	**% of Antibiotics**
Cephalosporin	***25.95***	***20.44***	***14.91***	***11.18***	***9.74***	***8.38***	***7.74***	***8.07***	***6.08***	***4.89***	***117.38***	***0.22***
Cefalexin	22.44	17.85	13.34	10.06	8.69	7.44	6.77	7.08	5.34	4.55	103.55	0.19
Cefradine	3.51	2.58	1.58	1.13	1.05	0.94	0.97	0.99	0.74	0.34	13.83	0.03
Lincosamides	***33.77***	***30.15***	***27.95***	***25.62***	***23.54***	***23.63***	***30.42***	***28.56***	***27.30***	***24.03***	***274.97***	***0.51***
Clindamycin	33.77	30.15	27.95	25.62	23.54	23.63	30.42	28.56	27.30	24.03	274.97	0.51
Macrolides	***306.16***	***273.66***	***240.78***	***210.24***	***179.32***	***157.59***	***173.65***	***146.83***	***126.15***	***112.30***	***1926.68***	***3.59***
Azithromycin	3.94	4.11	3.98	4.02	3.92	4.23	3.98	4.66	5.02	5.17	43.03	0.08
Clarithromycin	8.79	10.05	11.57	13.76	14.49	14.81	24.37	21.02	20.27	18.89	158.02	0.29
Erythromycin	293.42	259.51	225.23	192.46	160.91	138.55	145.29	121.16	100.86	88.24	1725.63	3.22
Nitroimidazoles	***1921.71***	***1774.96***	***1635.13***	***1521.35***	***1423.41***	***1357.16***	***1626.32***	***1466.30***	***1326.92***	***1204.89***	***15258.15***	***28.44***
Metronidazole	1921.71	1774.96	1635.13	1521.35	1423.41	1357.16	1626.32	1466.30	1326.92	1204.89	15258.15	28.44
Penicillins	***4493.35***	***4157.83***	***3852.91***	***3573.77***	***3325.04***	***3159.44***	***3715.12***	***3486.69***	***3213.82***	***2993.37***	***35971.35***	***67.04***
Amoxicillin	4406.86	4084.92	3791.47	3522.62	3283.02	3123.31	3675.20	3417.53	3137.72	2914.60	35357.25	65.90
Co-amoxiclav	36.09	33.99	31.51	28.01	24.27	21.43	24.34	22.73	20.62	18.62	261.61	0.49
Phenoxymethylpenicillin	50.40	38.92	29.94	23.14	17.75	14.70	15.59	46.43	55.48	60.15	352.50	0.66
Tetracyclines	***16.78***	***14.65***	***13.21***	***10.81***	***9.50***	***9.16***	***8.36***	***8.30***	***7.75***	***7.01***	***105.53***	***0.20***
Doxycycline	10.64	10.08	9.49	8.29	7.59	7.52	6.97	7.14	6.85	6.29	80.86	0.15
Oxytetracycline	1.70	1.36	1.38	0.88	0.70	0.62	0.56	0.53	0.39	0.35	8.47	0.02
Tetracycline	4.45	3.21	2.34	1.64	1.20	1.02	0.82	0.64	0.51	0.37	16.20	0.03
**Grand Total**	**6797.72**	**6271.69**	**5784.90**	**5352.98**	**4970.54**	**4715.36**	**5561.61**	**5144.76**	**4708.03**	**4346.48**	**53654.05**	**100.00**
**Analgesia**	**2014**	**2015**	**2016**	**2017**	**2018**	**2019**	**2020**	**2021**	**2022**	**2023**	**Grand Total**	**% of Analgesics**
Non-Opioid	***55.50***	***45.35***	***35.98***	***27.45***	***20.41***	***16.22***	***22.87***	***13.62***	***10.60***	***10.21***	***258.22***	***15.98***
Paracetamol	55.50	45.35	35.98	27.45	20.41	16.22	22.87	13.62	10.60	10.21	258.22	15.98
NSAIDs	***166.41***	***137.39***	***108.40***	***85.63***	***69.57***	***58.72***	***72.47***	***60.93***	***53.24***	***50.01***	***862.78***	***53.41***
Ibuprofen	147.11	118.95	91.94	69.73	54.75	44.43	39.59	35.82	30.50	28.88	661.68	40.96
Diclofenac	19.27	18.41	16.44	15.87	14.82	14.28	32.86	25.11	22.74	21.13	200.93	12.44
Opioid	***56.71***	***55.06***	***50.79***	***45.57***	***39.96***	***36.44***	***66.13***	***57.49***	***45.18***	***41.11***	***494.46***	***30.61***
Dihydrocodeine	56.71	55.06	50.79	45.57	39.96	36.44	66.13	57.49	45.18	41.11	494.46	30.61

Drug	Year (Items/100000 population)											
**Antibiotic**	**2014**	**2015**	**2016**	**2017**	**2018**	**2019**	**2020**	**2021**	**2022**	**2023**	**Grand Total**	**% of Antibiotics**
**Grand Total**	**278.62**	**237.80**	**195.18**	**158.65**	**129.94**	**111.39**	**161.48**	**132.05**	**109.03**	**101.33**	**1615.45**	**100.00**
Number of Dentists	23947	24089	24007	24308	24545	24684	23733	24272	24151	24193		
Population	54370300	54808700	55289000	55619500	55924500	56230100	56326000	56554900	57112500	57690300		
Dentists per 1000 population	44.04	43.95	43.42	43.70	43.89	43.90	42.14	42.92	42.29	41.94		

SI Table 1: Annual Prescribing Rates (items per 100000 population) of all analgesics and antimicrobials with percentage contribution over between 2014 and 2023. Note: Aspirin not included due to low number of prescriptions.

Substance<br>Antibiotic	Highest Value Month*	Highest Value (items per 100,000)	Feb 2020 Items per 100,000	Feb 2020 % Change from Peak	Dec 2023 Items per 100,000	Dec 2023 % Change from Peak	SUM CHI SQUARED Feb 2020 - Dec 2023 against peak month value	*P - Value*	Returned to Pre-Pandemic Levels?
Cephalosporin									
Cefalexin	Dec 2020	0.77	0.57	-26.5	0.49	-36.3	5.41	1.00	Return to or Below Pre-Pandemic Level
Cefradine	Jul 2021	0.10	0.07	-30.2	0.04	-65.1	1.11	1.00	Return to or Below Pre-Pandemic Level
Lincosamides									
Clindamycin	Jun 2020	3.09	1.83	-40.8	2.00	-35.3	10.70	1.00	Above Pre-Pandemic Level and Below Peak
Macrolides									
Azithromycin	Jun 2023	0.51	0.38	-25.0	0.41	-19.0	1.62	1.00	Above Pre-Pandemic Level and Below Peak
Clarithromycin	May 2020	2.82	1.14	-59.7	1.77	-37.3	20.05	1.00	Above Pre-Pandemic Level and Below Peak
Erythromycin	Jun 2020	13.36	10.18	-23.8	7.75	-42.0	66.57	**0.03**	Return to or Below Pre-Pandemic Level
Nitroimidazoles									
Metronidazole	Jun 2020	159.12	108.32	-31.9	96.17	-39.6	597.68	**0.00**	Return to or Below Pre-Pandemic Level
Penicillins									
Amoxicillin	Jun 2020	347.80	247.86	-28.7	242.03	-30.4	871.2	**0.00**	Return to or Below Pre-Pandemic Level
Co-amoxiclav	May 2020	2.22	1.74	-21.6	1.51	-32.2	4.92	1.00	Return to or Below Pre-Pandemic Level
Phenoxymethylpenicillin	Dec 2023	5.60	0.99	-82.4	5.60	0.0	47.68	0.44	Increased Use Post-Pandemic
Tetracyclines									
Doxycycline	Dec 2020	0.73	0.65	-10.9	0.56	-23.3	1.99	1.00	Return to or Below Pre-Pandemic Level
Oxytetracycline	Jul 2021	0.07	0.03	-53.9	0.02	-76.2	0.66	1.00	Return to or Below Pre-Pandemic Level
Tetracycline	Jun 2020	0.09	0.07	-22.6	0.02	-79.7	1.21	1.00	Return to or Below Pre-Pandemic Level

Analgesic	Highest Value Month*	Highest Value (items per 100,000)	Items per 100,000	% Change from Peak	Items per 100,000	% Change from Peak	SUM CHI SQUARED Feb 2020 - Dec 2023 against peak month value	P - Value	Returned to Pre-Pandemic Levels?
**Non-Opioid**									
Paracetamol	Mar 2020	3.71	1.20	-67.7	0.89	-76.1	84.50	**0.00**	Return to or Below Pre-Pandemic Level
**NSAIDs**									
Diclofenac	Jun 2020	4.86	0.96	-80.2	1.71	-64.8	76.46	**0.00**	Above Pre-Pandemic Level and Below Peak
Ibuprofen	May 2020	3.65	3.40	-7.0	2.24	-38.6	11.75	1.00	Return to or Below Pre-Pandemic Level
**Opioid**									
Dihydrocodeine	May 2020	7.32	3.22	-56.0	3.51	-52.0	62.93	0.06	Above Pre-Pandemic Level and Below Peak

SI Table 2. Comparison of Peak Prescribing Rate (items per 100,000 population) following the start of COVID-19 against the Prescribing Rate before the start of COVID 19 (Feb 2020) and latest prescribing rate (Dec 2023) *Aspirin Prescribing Rate too low to be included
